# A 6K-Deletion Variant of Salmonid Alphavirus Is Non-Viable but Can Be Rescued through RNA Recombination

**DOI:** 10.1371/journal.pone.0100184

**Published:** 2014-07-10

**Authors:** Tz-Chun Guo, Daniel X. Johansson, Øyvind Haugland, Peter Liljeström, Øystein Evensen

**Affiliations:** 1 Norwegian University of Life Sciences, Department of Basic Sciences and Aquatic Medicine, Oslo, Norway; 2 Department of Microbiology, Tumor and Cell Biology, Karolinska Institutet, Stockholm, Sweden; INRA, France

## Abstract

Pancreas disease (PD) of Atlantic salmon is an emerging disease caused by Salmonid alphavirus (SAV) which mainly affects salmonid aquaculture in Western Europe. Although genome structure of SAV has been characterized and each individual viral protein has been identified, the role of 6K protein in viral replication and infectivity remains undefined. The 6K protein of alphaviruses is a small and hydrophobic protein which is involved in membrane permeabilization, protein processing and virus budding. Because these common features are shared across many viral species, they have been named viroporins. In the present study, we applied reverse genetics to generate SAV3 6K-deleted (Δ6K) variant and investigate the role of 6K protein. Our findings show that the 6K-deletion variant of salmonid alphavirus is non-viable. Despite viral proteins of Δ6K variant are detected in the cytoplasm by immunostaining, they are not found on the cell surface. Further, analysis of viral proteins produced in Δ6K cDNA clone transfected cells using radioimmunoprecipitation (RIPA) and western blot showed a protein band of larger size than E2 of wild-type SAV3. When Δ6K cDNA was co-transfected with SAV3 helper cDNA encoding the whole structural genes including 6K, the infectivity was rescued. The development of CPE after co-transfection and resolved genome sequence of rescued virus confirmed full-length viral genome being generated through RNA recombination. The discovery of the important role of the 6K protein in virus production provides a new possibility for the development of antiviral intervention which is highly needed to control SAV infection in salmonids.

## Introduction

Salmonid alphavirus (SAV) is the causative agent of pancreas disease (PD) and sleeping disease in Atlantic salmon and rainbow trout, respectively. PD is a major problem in salmonid farming in Western Europe, causing high mortalities in the seawater stage. Diseased fish are clinically characterized by inappetence, fecal casts and emaciation with main pathological changes found in pancreas, heart and skeletal muscle [1]. To date, several subtypes of SAV sharing highly homogeneous genome sequences have been identified. Salmon pancreas disease virus (SPDV or SAV1) was first found in Ireland and Scotland in farmed Atlantic salmon [2]. Subsequently, sleeping disease virus (SDV or SAV2) which mainly affects rainbow trout was discovered in UK and France [3]. The third subtype of SAV (SAV3) is so far exclusively found in Norway affecting both Atlantic salmon and rainbow trout [4]. Additionally, another three discrete subtypes (SAV4–6) have been identified in Scotland and Ireland based on partial sequence (nsP3 and E2) analysis [5], and a marine SAV2-related virus is now also found in PD outbreaks in mid-Norway and Scotland [6]. All subtypes are geographically separated and distinguished based on phylogenetic analysis [7]. Only SAV 1–3 are fully sequenced, with a nucleotide identity of the three SAVs being above 90% over the entire genome.

SAV belongs to the genus alphavirus within the family *Togaviridae* [2]. Members of the alphavirus genus include many medically important pathogens such as Western equine encephalitis viruses (WEEV), Eastern equine encephalitis viruses (EEEV), Venezuelan equine encephalitis viruses (VEEV), Chikungunya virus, Ross River virus (RRV), Semliki Forest virus (SFV) and Sindbis virus (SINV). The diameter of an alphavirus virion is around 65–70 nm consisting of a tightly adherent lipid envelope surrounding an icosahedral capsid [8], [9]. The genome is a positive-sense, single-stranded RNA of approximately 11–12 kb which is capped at the 5′ end and polyadenylated at the 3′ end and thus serves directly as mRNA for translation of the viral replicase upon entry into host cells. The genomic organization of alphavirus is divided into two open reading frames (ORFs) [10], [11]. The first ORF encoding four non-structural proteins (nsP), designated nsP1-4, is responsible for transcription and replication of viral RNA. The second ORF, under the control of a 26S subgenomic promoter, codes for another polyprotein (capsid-PE2-6K-E1) making up the structural proteins (SP). PE2 and E1 are the envelope glycoproteins, associated as a heterodimer that migrates together with 6K through the secretory pathway to the plasma membrane. PE2 is cleaved to generate E3 and E2 glycoproteins in a post-Golgi compartment [12].

Alphavirus 6K protein is a small and highly hydrophobic protein. Alphavirus 6K together with several other viral gene products, such as Poliovirus 2B, Influenza A virus M2, HIV-1 Vpu and HCV p7, are classified as viroporins which share a common feature of enhancing membrane permeability [13]–[15]. The 6K proteins of SFV or SINV have been shown to be involved in membrane permeabilization at the late stage of infection [16]. Oligomerization of 6K proteins leads to ion channels formation in cell membranes and increases membrane permeability, which facilitates virus budding [15]. An identified aromatic domain (rich in aromatic amino acids) at the N-terminal of 6K shows a strong tendency to insert in the interfacial phase of the phospholipid bilayer which also facilitates membrane destabilization [9]. In addition to the role associated with membrane permeabilization, 6K also provides cleavage sites for polyprotein processing at its N-terminal and C-terminal ends. Both the E2-6K and 6K-E1 cleavage sites are found on the luminal side of ER and cleaved by signal peptidase [17]. A SFV mutant lacking the entire 6K is processed correctly between PE2 and E1 without altering glycoprotein formation, heterodimerization and intracellular transport [18]. However, the budding process of the SFV 6K deletion mutant is impaired and virus titer is reduced [19]. Deletion of 6K in SINV also causes impaired budding and in addition the cleavage of polyprotein becomes less efficient [20]. The sequence of SAV3 6K has been identified but protein function and its role in virus formation have not been studied in any detail. We wanted to explore if deletion of the SAV3 6K gene would attenuate virus infectivity and thus could represent a strategy for development of a live-attenuated viral vaccine against PD. As the cleavage sites of the polyprotein including 6K remain to be proven experimentally for salmonid alphaviruses, these were deduced from amino acid sequence homology with other alphaviruses [21]. We examined the role of the 6K protein by deleting the entire gene using a reverse genetics approach and then studied generation of infectious progeny *in vitro*.

## Materials and Methods

### Cell culture and virus

Chinook salmon (*Oncorhynchus kituch*) embryonic cells (CHSE-214) were obtained from the American Type Culture Collection (ATCC). Chum salmon (*Oncorhynchus keta*) heart cells (CHH-1) and Bluegill fry cells (BF-2) were obtained from the European Collection of Cell Cultures (ECACC). All cell lines were maintained at 20°C in L-15 media (Invitrogen) supplemented with 5% FBS (Invitrogen), 2 mM L-glutamine with or without 50 µg/mL gentamycin. The SAV3 isolate (SAV3-H10) used in this study was obtained from a clinical outbreak of pancreas disease (PD) in Atlantic salmon from western Norway [22]. The isolate was propagated by inoculating homogenized heart tissue samples onto 80% confluent CHSE-214 cells maintained in growth medium supplemented with 2% FBS at 15°C. The isolate was confirmed as SAV3 by RT-PCR and DNA sequencing.

### SAV3-H10 full genome sequencing

Virus was propagated in CHSE-214 cells until passage 11 at which time full genome sequencing was performed. Virus was harvested at nearly full CPE. The supernatant was first cleared by centrifugation at 1500 rpm for 10 minutes before virus sedimentation was performed by ultracentrifugation (Beckman, Optima Ultracentrifuge) in a volume of 1.5 mL with rotor TLA-45 at 65,000×g for 1.5 hours. After ultracentrifugation, most of the supernatant was carefully removed and discarded and the remaining 150 µl containing the virus pellet was subjected to viral RNA isolation using the QIAamp Viral RNA kit according to the manufacturer’s instructions (Qiagen). cDNA synthesis was performed using the Transcriptor First Strand cDNA Synthesis Kit (Roche) with the mixture of random hexamers (1 µl) and oligo dT primers (1 µl). PCR was performed using Phusion High-Fidelity DNA Polymerase (Finnzymes) with primers (Table 1) based on a previously published sequence (GenBank no. AY604238). The rapid amplification of cDNA ends (RACE) technique was performed to obtain the full-length sequence including the exact 5′ and 3′ ends. The procedure was performed according to the manufacturer’s instructions (BD SMART RACE cDNA Amplification Kit, Clontech). 5′ RACE-GSP and 3′ RACE-GSP primers were designed based on sequence information obtained in the first round (Table 1). RACE products were purified and subcloned into pGEM-T Easy vector, and for both 5′- and 3′-RACE, 10 colonies were picked up for sequencing (Eurofins MWG).

**Table 1:** Primers used for genome sequencing and vectors construction.

*Used for*	*Name*	*Primer sequence (5*′*→3*′*)*	*Orientation*	Restriction site
SAV3-H10 genome sequencing except 5′ and 3′ end	F1	TCACTGTAGATTTGCCCGCGG	For	
	R1	CCAACAGGTGTTACGCTTCCCGT	Rev	
	F2	ATAATGAGCTCATGACTGCGGCTGC	For	
	R2	GCTTCAGCACTGTGACCCGTTCC	Rev	
	F3	ACGGAGACGCTGTCCAGTTTCG	For	
	R3	TACACGGGGAAGGTGCTCTGTC	Rev	
	F4	AAGTGGAAAGCTGGTACAGAGTGGG	For	
	R4	GCACTTCTTCACCACGCAGTAGGTAA	Rev	
5′ RACE primer	5′ RACE-GSP	TGGAACACGAACGGCTCGAACCCGATCC	Rev	
3′ RACE primer	3′ RACE-GSP	CGCTTGGTGAAGTGGTGACGGCAGTCC	For	
Full-length SAV3-H10 cDNA clone construction	P1	GCTTGATATCGAATTCGATAAATCCAAAAGCATA	For	*EcoRI*
	P2	ACCGCGGTGGCGGCCGCAACGAGCTTAAGGTGGGG	Rev	*NotI/AflII*
	P3	CATGAATTCTAACTACCCCACCTTAAGCTCGTTCGGAGT	For	*EcoRI/AflII*
	P4	TTGCGGCCGCCTTATATTGAAAATTTTAAAACCAATAGATGACTCA	Rev	*NotI*
	P5	AAAAGCGGCCGCGCTAGC(T)_60_CTTATATTGAAAATTTTAAAACCA	Rev	*BmtI/NotI*
Modification of full-length cDNAat 5′ end andconstruction ofΔ6k and helpercDNA clones	T7-HH-F	TAATACGACTCACTATAGGGTGGATTTATCCTGATGAGT CCGTGAGGACGAAACTATAGGAAAGGAATTCCTATAGT CGATAAATCCAAAAGCATACA	For	
	CMV-R	GATCTGACGGTTCACTAAACC	Rev	
	Δ6k-F	TACGAACACACCGTGGTGGTCCCAATGGA	For	
	Δ6k-R Helper-FHelper-R	CGCACGAGCCCCAGGTATGCAGCACAATG TGTAAACCATCTGCCGTTAGCCAC TGATATATGTATGCTTTTGGATTTATCGA	Rev For Rev	
Construction ofSFV-REP/SAV3-SP	CS-F	AAAACCCGGGATGTTTCCCATGCAATTCAC	For	*XmaI*
	E1-R	AAAAACTAGTTTAGCTCTTGACTATCCGGATTC	Rev	*SpeI*

### Construction of a full-length SAV3 cDNA clone

**Figure 1:**
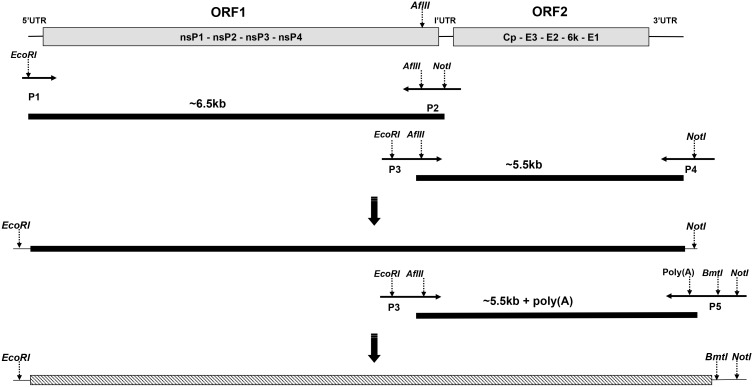
Schematic illustration of the full-length SAV3 cDNAs constructed. AflII is a unique restriction site residing within SAV3/P2 and P3/P4, respectively. The purified PCR products were subcloned into the pBluescript vector with *EcoRI* and *NotI* sites. The 5.5 kb fragment was thereafter subcloned into the pBluscript vector containing the 6.5 kb fragment vector at *AflII* and *NotI* sites, to make the full-length SAV3 cDNA construct without poly(A). Primer P5 containing poly(A) was used in combination with primer P3 to introduce poly(A). The final insert constituting full-length SAV3 cDNA including poly(A) was finally subcloned into pTurboFP635-N at *EcoRI* and *NotI* sites. Fragments were inserted in pBluescript vector (solid, black line) and in pTurboFP635-N (hatched line).

Viral RNA isolation and cDNA synthesis were performed as described above. The infectious clone was constructed by combining two clones containing roughly half of the genomic cDNA (Figure 1). The first fragment (6374 bp) was amplified with primers P1 and P2 flanked with *EcoR* I and *Afl* *Π*/*Not* I restriction sites respectively (Table 1). The second fragment (5527 bp) was amplified with primers P3 and P4 flanked with *EcoR* I/*Afl* *Π* and *Not* I sites respectively. PCR reactions contained 28.5 µl H_2_O, 10 µl 5X Phusion HF Buffer, 3 µl 10 mM dNTPs, 6 µl 0.5 µM forward plus reverse primers, 2 µl viral cDNA and 0.5 µl Phusion High-Fidelity DNA Polymerase (Finnzymes). PCR was performed using the following conditions: 98°C 30 s, 35 cycles of 98°C 10 s, 60°C 30 s, 72°C 4 min, and finally 72°C 5 min. The two fragments constituting the entire viral genome were cloned separately into the pBluescript vector (Stratagene) at *EcoR* I and *Not* I sites following standard cloning procedures. pBluescript vectors containing the 6.5 kb and 5.5 kb fragments were subsequently digested with *Afl* *Π* and *Not* I and purified, before the full-length SAV3 cDNA clone without poly(A) was constructed by combining the two fragments at *Afl* *Π*/*Not* I site (Figure 1). A poly(A) tail was added by PCR at the 3′ end of the cDNA clone using primer P5 containing the poly(A) tail and flanked by *Bmt*I and *Not*I sites in combination with primer P3 (Table1). The PCR product was gel purified and combined with the pBluescript vector containing the 6.5 kb fragment at the *Afl* *Π*/*Not* I sites to yield the full-length SAV3 cDNA clone with poly(A). The resulting infectious cDNA clone was finally transferred from the pBluescript backbone and inserted into the pTurboFP635-N vector (Evrogen) at the *EcoR*I and *Not*I sites, and designated as pSAV3-FL.

### Modification of the 5′ end, deletion of the 6K gene and generation of helper cDNA vector

To ensure precise cleavage at the 5′ end during transcription, a hammerhead (HH) ribozyme sequence [23] was inserted immediately upstream of the 5′ UTR region of the full-length cDNA construct. Furthermore, a T7 promoter was fused upstream to the HH sequence to obtain the capability of *in* *vitro* transcription. This was achieved by long-range PCR using the Phusion system as described above, with primers T7-HH-F and CMV-R (Table 1) and *Not*I linearized pSAV3-FL DNA as template. The obtained PCR product was purified and treated with a T4 polynucleotide kinase (Promega) and subsequently self-ligated with T4 ligase (Promega). The full-length SAV3 cDNA construct containing the T7 promoter and HH sequences was named pSAV3-HHFL. Deletion of the 6K gene was obtained by amplifying pSAV3-HHFL using PCR with appropriate outward-facing primers (Δ6K-F and Δ6K-R), creating a PCR product of the entire plasmid but lacking the gene of interest (Table 1). The PCR product was turned into a plasmid as described above and designated pSAV3-HHΔ6K. A SAV3 helper cDNA vector was constructed by deleting the replicon (nsP1, nsP2, nsP3, and nsP4) from the full-length cDNA clone except for 100 nt at carboxy-terminal of nsP4 that was preserved. This was achieved by PCR using the primers Helper-F and Helper-R (Table 1). The sequences of all final constructs were confirmed by DNA sequencing (Eurofins MWG).

### Generation of polyclonal antibodies against SAV3 structural proteins (mouse anti-SAV3-SP)

The DNA vector used for immunization (pSFV-REP-SAV-SP) was constructed by inserting the structural protein genes of SAV3-H10 (SAV3-SP) amplified by PCR using the primers CS-F and E1-R (Table 1) into the Semliki Forest virus (SFV) replicon system [24]. Five Balb/C mice were immunized with pSFV-REP-SAV-SP (10 µg DNA/20 µl PBS per mice) via intradermal injection near the base of the tail followed by intradermal electroporation (Cyto Pulse Sciences, Inc., Glen Burnie, MD) as previously described [25]. DNA immunization was repeated using the same DNA vector at 3 and 10 weeks after the first injection. Additionally a booster immunization with recombinant SAV3-E2 protein (10 µg/50 ul PBS) expressed in E. coli [22] and mixed with 50 µl Freund’s incomplete adjuvant (Sigma F5506) were given intramuscularly divided equally between the hind legs at week 6. Sera from the five immunized mice and two non-immunized controls were then harvested at week 13. After confirming specific staining in SAV3 infected cells by immunofluorescence (IF) assays, sera from all mice were pooled and stored at −20°C. All mice were bred and housed at the animal facility of the Department of Microbiology, Tumor and Cell Biology at the Karolinska Institutet, Stockholm, Sweden. Mice were anaesthetized with 4% isoflurane (Baxter Medical AB, Kista, Sweden) during all intradermal injections and electroporations. All mice experiments were approved by the Committee for Animal Ethics in Stockholm, Sweden, and performed according to given guidelines. Another antibody used in this study, rabbit anti-SAV3-E2, was generated previously [22].

### Determination of anti-SAV3-SP IgG levels in immunized mice

ELISA plates (Nunc-immuno plate MaxiSorp F96) were coated with recombinant SAV3-E2 protein diluted in PBS. 50 µl of E2 protein (10 µg/mL) was added into each well and the plates were incubated at 4°C overnight. The plates were washed three times with 250 µl of phosphate-buffered saline with 0.1% Tween20 (PBST) and then blocked with 5% non-fat dry milk diluted in PBST for 2 hours at room temperature. Washing was performed as described above. Two-fold serial dilutions of sera in PBST (1∶20 to 1∶40960) from individual mouse (1–5) were then added to the wells and incubated for 1 hour at room temperature. The wells were subsequently washed and incubated for another 1 hour at room temperature with 100 µl of a 1∶1000 dilution of peroxidase-conjugated goat anti-mouse IgG (Dako), followed by color development with 1,2-phenylenediamine dihydrochloride (OPD) tablets (Dako). 100 µl OPD solution was added to each well and plates were incubated for 30 min at room temperature before enzyme reactions were stopped by addition of 0.5 M H_2_SO_4_. Optical density (OD) was read as absorbance at 490 nm by an ELISA plate reader.

### In situ detection of SAV3 by immunohistochemistry

Sections were made from paraffin embedded tissue of pancreas from SAV3 infected fish and blocked with 5% bovine serum albumin (BSA) for 20 min before incubating with mouse anti-SAV3-SP antibody at a dilution of 1∶1000 for 30 min at room temperature. This was followed by the secondary antibody (biotinylated anti-mouse immunoglobulin diluted 1∶300) for 30 min at room temperature. After incubation with Streptavidin–biotin alkaline phosphatase (1∶500 dilution) for 30 min, sections were stained with Fast Red substrate (Sigma) and counterstained with hematoxylin.

### Immunofluorescence (IF) assay

Detection of SAV3 viral proteins in cultured cells was performed by IF assay. Virus infected cells and cells transfected with SAV3 cDNA clones were fixed in 4% formaldehyde for 15 min at room temperature followed by permeabilization with 0.1% Triton-X100 for 5 min on ice. Cells were then blocked with 5% non-fat dry milk (Bio-Rad) for 30 min before incubated sequentially with the primary and secondary antibodies. Cells were incubated at room temperature for one hour with primary antibody (mouse anti-SAV3-SP) diluted 1∶1000 in 5% BSA. After washing four times with PBS, secondary antibody diluted 1∶400 in 5% BSA (Alexa Fluor 488- goat anti-mouse IgG, Invitrogen) was incubated for another 30 min at room temperature. Cells were again washed four times with PBS and counterstained with Hoechst 33324 (5 µg/ml) for 2–5 minutes at room temperature before washing with PBS and analyzed using a fluorescence microscope (Olympus, IX81).

### Transfection of cells and recovery of recombinant virus in three susceptible cell lines

CHSE-214, CHH-1 and BF-2 cells grown to 80% confluence in 24-well plates were transfected using the Fugene HD transfection reagent (Roche) following the manufacturer’s protocol. Briefly, 2 µg of each SAV3 infectious cDNA clone was diluted in 100 µl Opti-MEM I reduced serum medium (Invitrogen) before 6 µl of transfection reagent was added. The transfection mix was incubated at room temperature for 15 min before 25 µl was added into each well. Transfected cells were incubated at 15°C and harvested at 2, 4, 7, 10 and 13 days post infection. The collected virus supernatant was subjected to virus titration by TCID_50_ in CHSE-214 cells using the Spearman–Kärber method [26].

### Real-time PCR

Real-time PCR was performed using the LightCycler LC480 instrument and LightCycler 480 SYBR Green I Master mix (Roche) in a final volume of 20 µl. The PCR reactions were first incubated at 95°C for 10 min, followed by 40 amplification cycles (10 s at 95°C, 20 s at 60°C and 8 s at 72°C). Final concentration of primers in the reaction mix was 0.5 µM and sequence information of primers used to assess *in* *vitro* expression of IFNα, Mx, and ISG15 were as previously described [22]. The specificity of the PCR product from each primer pair was confirmed by melting curve analysis and subsequent agarose gel electrophoresis. The 2^–△△^ *C*T method was used to calculate expression levels as described elsewhere [27]. 2^–△△^ *C*T is the relative mRNA expression representing fold induction over the control group. All quantifications were normalized to β-actin (endogenous gene). Statistical analyses and graphs were performed with the help of GraphPad Prism 5.0 (GraphPad software Inc., USA).

### Total protein analysis and radioimmunoprecipitation assay (RIPA)

CHH-1 cells were seeded in the 6-well plate one day prior to infection with wt SAV3 or transfection with either pSAV3-HHFL or pSAV3-HHΔ6K cDNA clones. Virus infected cells were incubated at 15°C for 48 hours while transfected cells were incubated for 96 hours. Cells were then starved in methionine-free MEM medium (Mpbio) for 30 minutes followed by radiolabeling with 50 µCi [^35^S] methionine/ml (Montebello Diagnostics) in starvation medium for 6 hours prior to harvest. For total proteins analysis, cells were lysed using CelLytic™ M solution (Sigma) and clarified by centrifugation as described in the manufacturer’s manual. 15 µg of total proteins was loaded per lane (wt SAV3 infected cells, pSAV3-HHFL transfected cells, pSAV3-HHΔ6K transfected cells, and non-treated control cells), separated by SDS-PAGE and blotted onto a PVDF membrane (Bio-Rad). The membrane was exposed in a phosphorImager cassette for at least 16 hours before image was scanned using the Typhoon imager (GE healthcare). For the analysis of membrane proteins, cells were lysed with phase separation lysis buffer and separated into membrane-bound detergent phase and soluble phase following a protocol described previously [28]. Proteins in the detergent phase were diluted 50-fold in PBS containing 1% Triton X-100 and a protease inhibitor cocktail (Sigma) before being added to pre-incubated complex of IP matrix (Santa Cruz) and mouse anti-SAV3-SP antibodies. The protein-antibody-IP mixture was incubated overnight at 4°C on a rotator. The reaction mixes were then carefully washed three times with PBS, resuspended in protein buffer and boiled for 5 minutes. Protein samples were then analyzed on SDS-PAGE, blotted and scanned as described above.

## Results

### Construction of an infectious SAV3 cDNA clone

**Figure 2:**
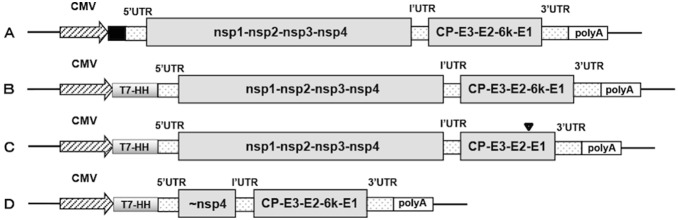
Organization map of full-length SAV3 and deletion variants constructed. (A) Full-length SAV3 cDNA cloned in pTurboFP635-N vector without modifications. (B) Full-length SAV3 cDNA with modification at the 5′ end. Non-viral sequences (█) were removed and a T7 promoter was inserted right after the CMV promoter. In addition, a hammerhead ribozyme (HH) sequence was added in front of 5′ UTR in order to generate the accurate 5′ end. (C) SAV3 Δ6k mutant: the entire 6k gene was deleted (▾) from the full-length construct (B) by PCR. (D) Organization of the helper cDNA vector containing sequences starting about 100 bases upstream of the nsP4 C-terminal throughout the structural proteins including poly(A).

The isolate (SAV3-H10) used in this study has previously not been fully characterized. With the purpose to obtain a functional infectious cDNA clone, the first step was to determine the full genome sequence including the 5′ and 3′ UTR regions by RACE. Genome sequencing except for the 5′ and 3′ end was performed with primers based on known sequences from other SAV3 strains deposited in GenBank. To ensure functionality of the recombinant virus, the exact sequences of the viral ends were revealed by 5′ and 3′ RACE. To obtain the full-length SAV3 cDNA construct, two fragments with the sizes of 6374 bp and 5527 bp was combined, using the internal restriction site *AflII* and external restriction sites *EcoR I* and *Not I* for ligation (Figure 1). At this stage the full-length SAV3 cDNA excluding 3′ poly(A), 11877 bp in length, was inserted into the pBluescript vector. The addition of a poly(A) tail sequence at the 3′ end was made by PCR and the resulting full-length SAV3 cDNA containing poly(A) was subcloned into the pTurboFP635-N vector (Figure 2A). Two different cDNA clones were fully sequenced and the sequencing result showed nucleotide sequences identical to the parental virus sequences. This suggests an efficient and predictable method for construction of full-length SAV cDNA clones has been established. In order to ensure precise cleavage at the 5′ end during transcription, a HH ribozyme sequence was inserted immediately upstream of the 5′ UTR as described by others [23]. The construct was further engineered by fusing a T7 promoter in front of the HH sequence. Finally, the non-viral sequences between the CMV and T7 promoter were removed, generating the final infectious cDNA clone (Figure 2B) for rescue of recombinant virus.

### Production and characterization of polyclonal antibodies against SAV3 structural proteins

**Figure 3:**
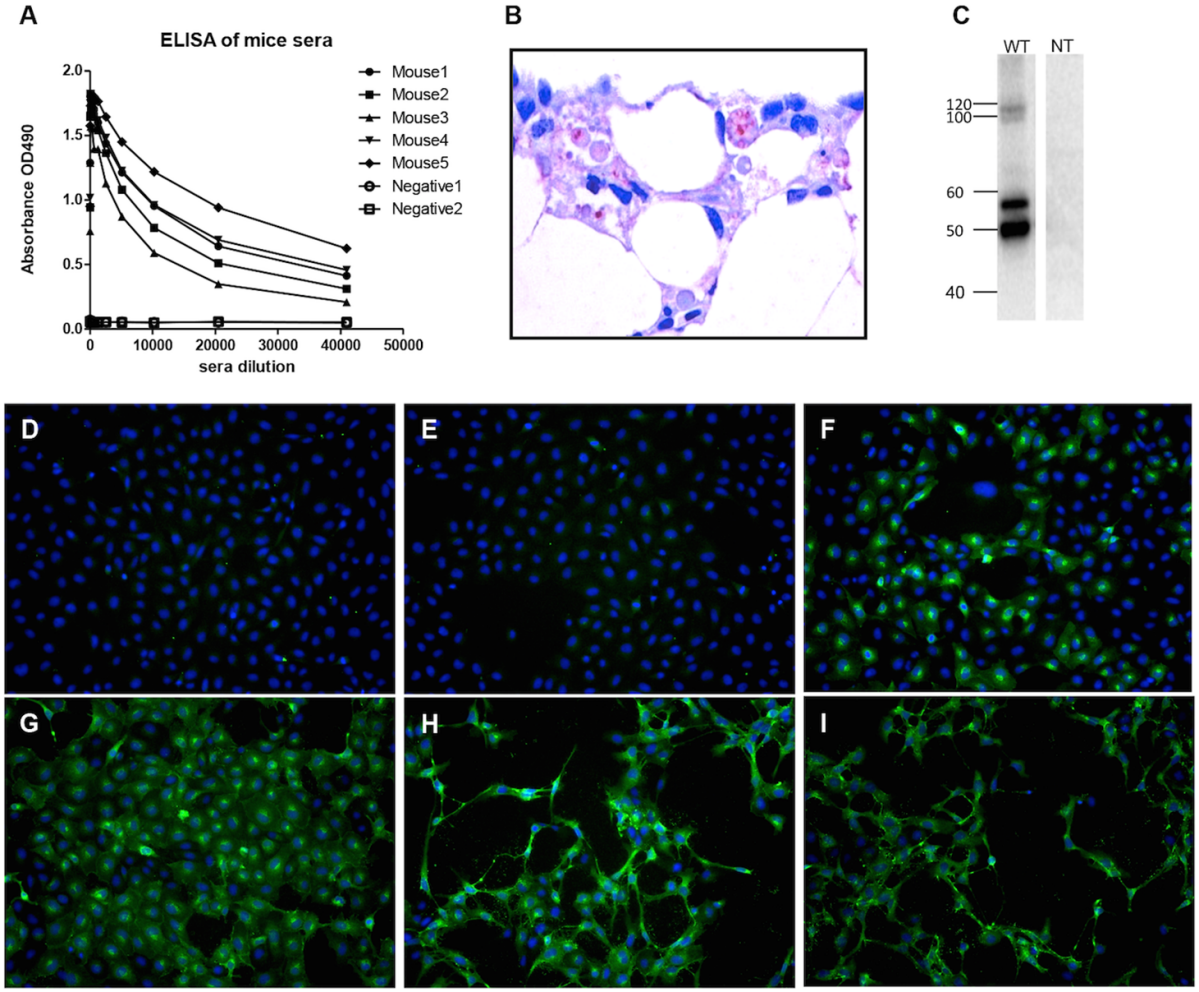
Characterization of the mouse anti-SAV3-SP antibody against structural proteins (SP). (A) All mice (no. 1–5) immunized with pSFV-Rep-SAV3-SP yielded high titer antibodies by ELISA. (B) Detection of SAV3 by immunohistochemistry in necrotic, exocrine pancreas cells of Atlantic salmon infected with wild-type SAV3. (C) Detection of SAV3 viral proteins by western blot. WT: wild-type SAV3. NT: Non-treated cells used as negative control. (D–I) Dynamics of SAV3 viral protein expression by immunostaining in CHSE-214 cells at different time points post infection using wild-type SAV3 (MOI = 1). (D) 16 hpt (E) 24 hpi (F) 40 hpi (G) 48 hpi (H) 72 hpi (I) 96 hpi.

To evaluate the functionality of the constructed full-length cDNA clone and modified constructs, specific anti-SAV3 antibodies are needed. The SFV replicon system has proven to be a useful vector for expression of heterologous genes and the vector design for enhanced immunogenicity in mice [24], [29] was employed to generate anti-SAV3 antibody. ELISA results confirmed high levels of antibodies generated from immunized mice with minimal background, while non-immunized mice were negative (Figure 3A). The anti-SAV3 mice serum also allowed specific detection of SAV3 in infected pancreatic tissue by immunohistochemistry (Figure 3B). When mouse anti-SAV3-SP serum was incubated with proteins from wt SAV3 infected CHH-1 cells blotted on a PVDF membrane, four bands were clearly revealed. No specific bands were detected in uninfected control cell samples (Figure 3C). Two bands at around 50 kDa and 57 kDa were most likely E2 and pE2 (E2+E3), respectively, although we cannot rule out that one of them might as well be E1. Additionally, two larger bands with the size of around 100 kDa and 110 kDa were possibly corresponding to uncleaved polyproteins. Further analysis revealed that the antibody provided strong and highly specific detection of SAV3 in cell culture by immunostaining, making it possible to evaluate the expression kinetics of SAV3 viral proteins during the course of viral infection. As shown in Figure 3D–I, following inoculation with wt SAV3, no viral protein was detected at 16 and 24 hours post inoculation (hpi), while some cells expressed viral proteins at 40 hpi. At 48 hpi, an increasing number of positive staining cells were detected concomitant with a slight change in morphology. CPE consisting of cell shrinkage was observed from 72 hpi, progressing into more severe CPE being observed at 96 hpi.

### rSAV3 is predominantly recovered in CHH-1 which have a feeble IFN response

**Figure 4:**
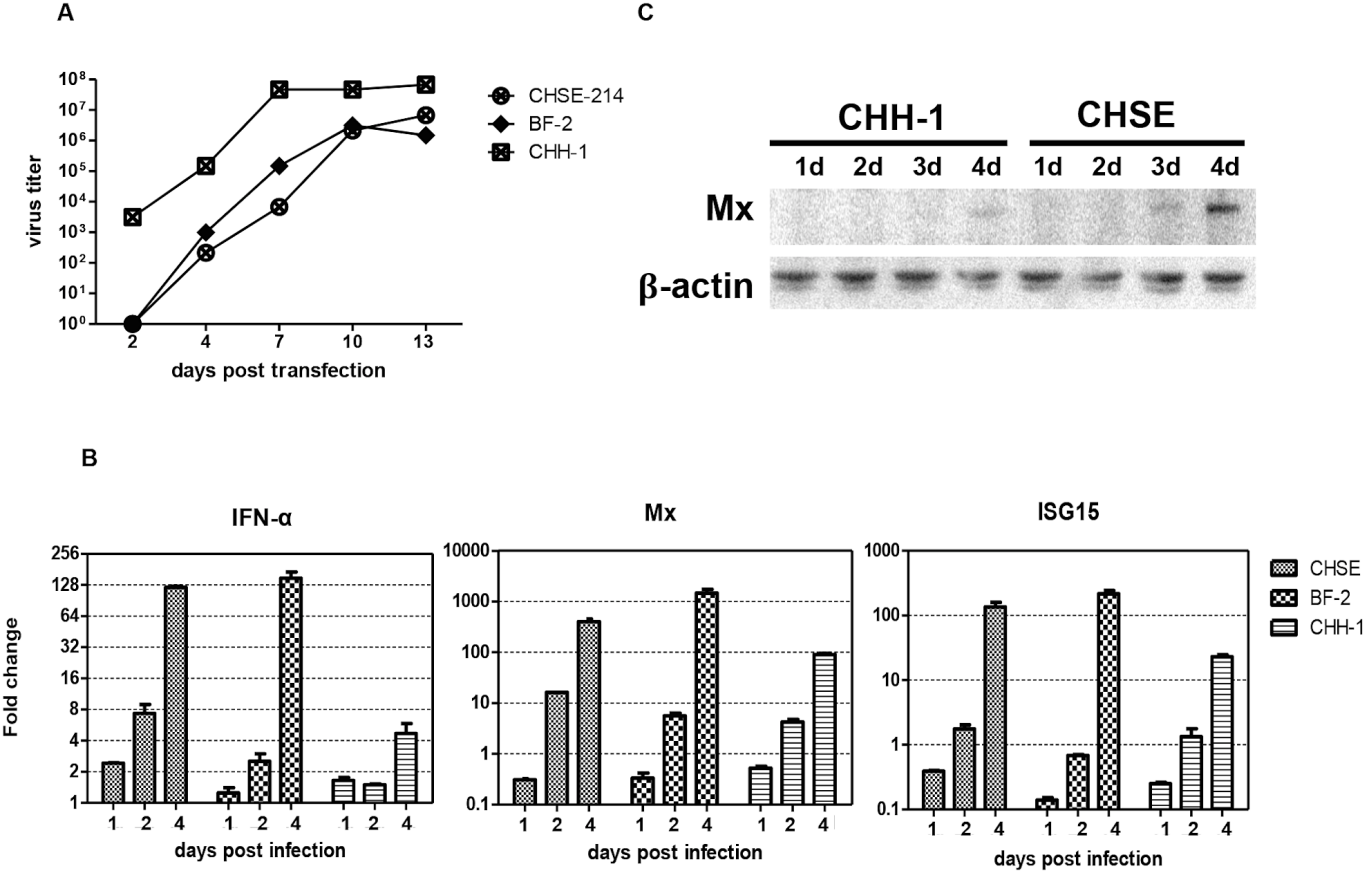
Recovery of rSAV3 in three susceptible cell lines and detection of IFN and ISGs response in each cell line. (A) The constructed infectious SAV3 cDNA clone was transfected into CHH-1, CHSE-214, and BF-2 cell lines. Supernatant from each cell line was collected at indicated time points and subjected to virus titration by TCID_50_. (B) Elicited IFN and ISG mRNA expression (Mx and ISG15) following SAV3 infection (MOI = 1) was evaluated by real-time PCR. Each time-point represents three biological replicates. (C) Expression of the Mx protein was detected in CHH-1 and CHSE-214 cell lines by western blot at 1 to 4 days post infection with wtSAV3 (MOI = 1). β-actin expression was used as internal control.

In order to rescue infectious SAV3 virus by reverse genetics in a virus-favored environment, we first sought for an optimal cell line for virus replication. The full-length cDNA clone was transfected into three susceptible cell lines, CHSE-214, BF-2, and CHH-1. Extensive CPE was observed in CHH-1 cells appearing at 5 days post transfection (dpt). Comparable CPE was observed at 10 dpt in CHSE-214 and BF-2 cells (data not shown), suggesting that these cell lines were more resistant to SAV3 infection. In line with these results, infectious virus was rescued as early as 2 dpt in CHH-1 cells and increased to a viral titer of 4.7×10^7^ TCID_50_/ml at 7 dpt and 6.8×10^7^ TCID_50_/ml at 13 dpt (Figure 4A). The rescue of infectious virus was significantly delayed in CHSE-214 and BF-2 cells (rescued from 4 dpt) and the resulting peak titers were 1–1.5 log_10_ lower than what was obtained in CHH-1 cells (Figure 4A). In a previous study we have demonstrated the effect of IFN-α on limiting SAV3 viral replication [22] and this prompted us to associate the discrepancy of CPE and viral replication with capabilities of IFN induction in different cell lines. We therefore measured innate immune responses in these three cell lines post SAV3 infection. The induction of IFNα and two interferon-stimulated genes (ISGs), Mx and ISG15, was quantified at RNA expression level by real-time PCR. Indeed, the result showed that responses in both IFNα and the two ISGs were low in CHH-1 while significantly stronger responses were detected in CHSE-214 and BF-2 cells (Figure 4B). At 1 and 2 dpi, the induction of IFNα and ISGs was in general low in all examined cell lines. At 4 dpi, markedly high levels of IFNα and ISGs were induced in CHSE-214 and BF-2 cells (Figure 4B). IFNα was upregulated ∼120-fold in CHSE-214 and ∼150-fold in BF-2 cells. In contrast, upregulation of IFNα was only 5-fold in CHH-1 cells. For the ISGs, Mx was upregulated ∼400-fold in CHSE-214, ∼1450-fold in BF-2, and 90-fold in CHH-1 cells, while ISG15 was upregulated ∼135-fold in CHSE-214, 215-fold in BF-2 cells, and 23-fold in CHH-1 cells. The same pattern was obtained for Mx protein expression in CHSE-214 and CHH-1 cells by western blot (Figure 4C), confirming differential induction of IFN responses in different SAV3 susceptible cell lines. From the results above, we conclude that CHH-1 is the optimal cell line for rescue of infectious SAV3 virus from full-length cDNA clones, and thus the following studies were performed in CHH-1 cells.

### SAV3 6K contains a unique insertion not found in other alphaviruses

**Figure 5:**
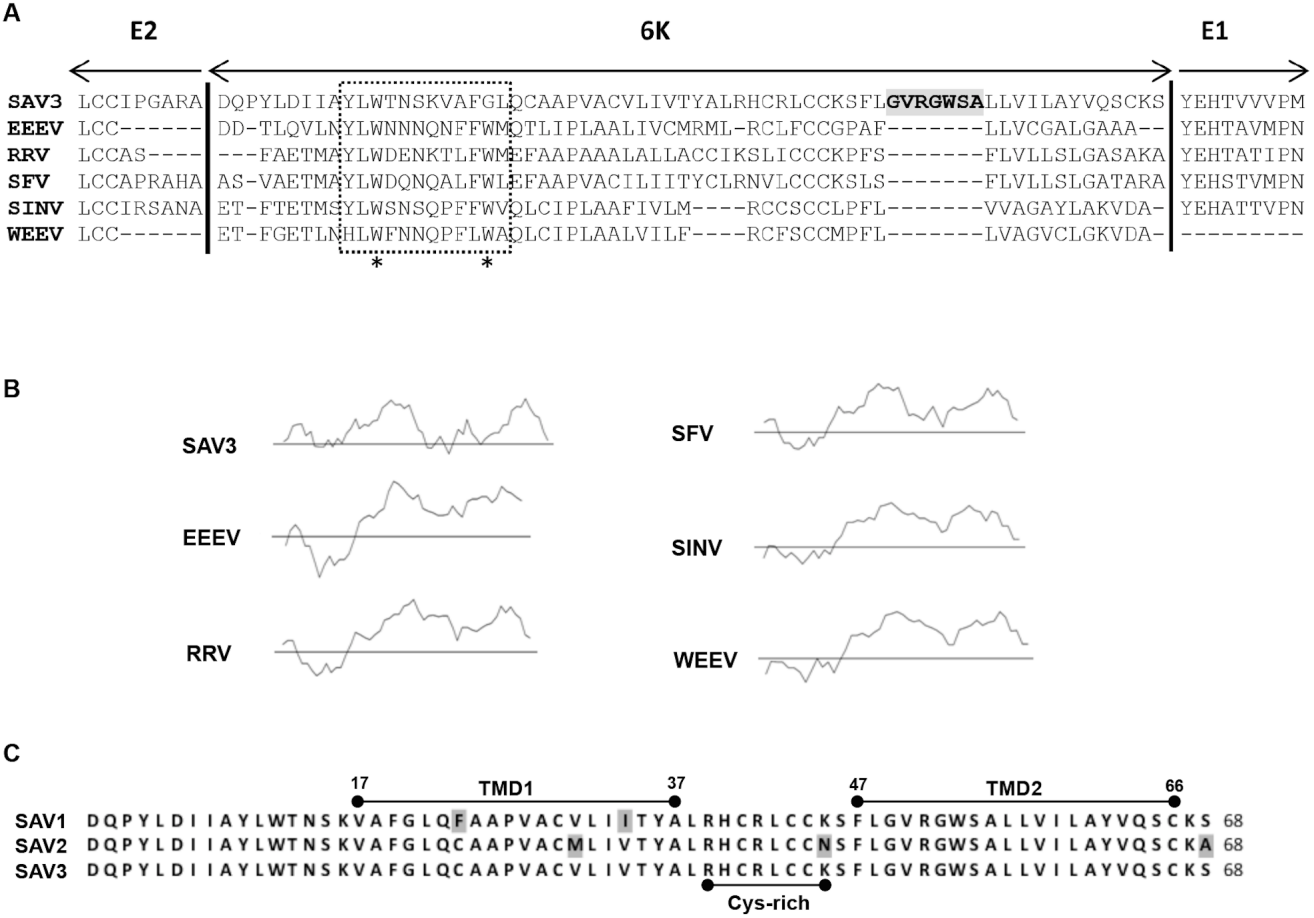
Analysis of SAV3 6K protein. (A) The SAV3 6K protein sequence aligned with other alphaviruses. The C-terminal end of E2 and N-terminal end of E1 were included to show the conservation around the (E2-6K) and (6K-E1) signal peptidase cleavage sites. The aromatic domain is shown inside the rectangular square. The unique seven amino-acid domain in SAV, GVRGWSA, is highlighted in grey. EEEV: Eastern Equine Encephalitis; RRV: Ross River; SFV: Semliki Forest; SINV: Sindbis; WEEV: Western Equine Encephalitis. (B) Hydrophobicity scale analysis of 6K amino acids by Kyte-Doolittle plots. The central line is the hydrophobicity scale at zero. Above zero are hydrophobic regions. (C) SAV1–3 6K protein sequences were aligned and two transmembrane domains were predicted. Between two TMDs, there is a cysteine rich domain.

The 6K protein of SAV3 or other salmonid alphaviruses has not been characterized in any detail. In the present study, we first aligned the SAV3 6K sequence with sequences from other alphaviruses (Figure 5A) and performed hydrophobicity scale characterization (Figure 5B). Results showed that SAV3 6K shares 38–51% similarity to other alphaviruses, while being highly conserved when compared to SAV1 (94.1%) and SAV2 (95.1%). Although sequence similarities were found, differences between SAV and other alphaviruses were detected. First, the 6K protein of SAV3 possesses 68 amino acids, being slightly larger than 6K proteins of other alphaviruses, manifested with a unique insertion of 7 amino acids (GVRGWSA) located in the transmembrane domain 2 (TMD 2) (Figure 5A). Second, two hydrophobic residues in the aromatic domain of 6K, Trp-11 and Trp-19, are fully conserved among all alphaviruses (Figure 5A). The first corresponding residue in SAV3 6K, Trp-12, is conserved with other alphaviruses. However, the second corresponding hydrophobic residue in SAV3 6K is Gly-20. Hydrophobicity analysis of the 6K protein using the Kyte-Doolittle method, showed two potential transmembrane domains (TMD) in SAV3, as seen in other alphaviruses (Figure 5B). These two putative TMDs were further analyzed by TMpred program [30]. The first TMD starts from Val-17 to Leu-37, with a predicted outside to inside orientation and the second TMD is from Phe-47 to Cys-66, with the orientation inside to outside membranes (Figure 5C). A cysteine rich domain between the two TMDs was found in SAV3 and conserved with other alphaviruses, possibly forming a cytosolic loop connecting the two transmembrane domains of 6K. Aligning 6K among SAV subtypes 1–3 showed that only five amino acids were not conserved between them (Figure 5C), while the aromatic domain and the unique insertion (GVRGWSA), were conserved among three SAV subtypes.

### SAV3 lacking the entire 6K is deficient in producing infectious particles

**Figure 6:**
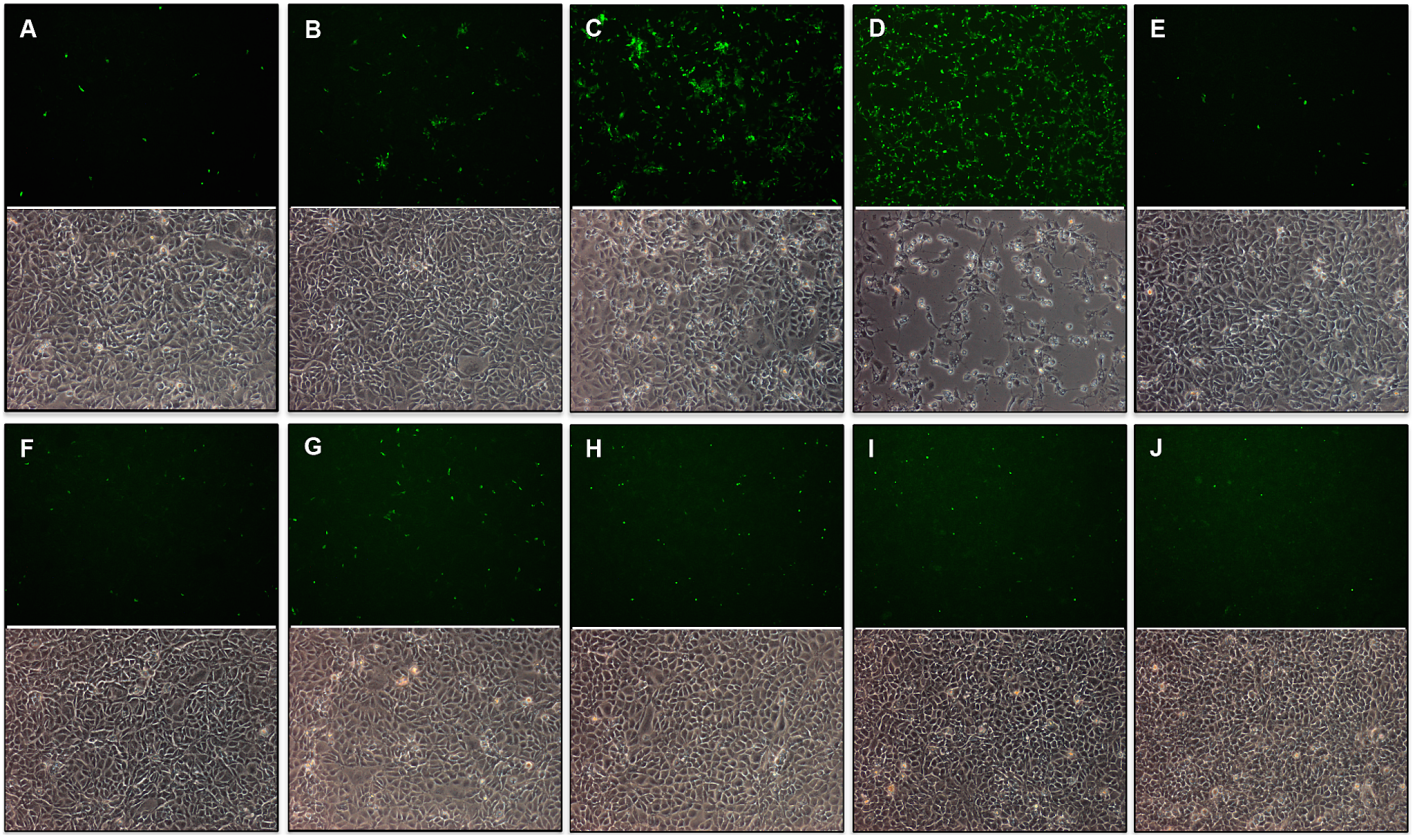
Immunostaining showing expression of viral proteins in CHH-1 cells. Cells transfected with pSAV3-HHFL clone were stained at 2, 3, 4, 6 dpt. (A–D), while cells transfected with the pSAV3-HHΔ6K construct were stained at 2, 3, 4, 6, 8, 10 dpt (E–J). Cell morphology at each corresponding time point is shown below the fluorescence photo.

We then went on to investigate the role of 6K for production of viable progeny by constructing recombinant SAV3 lacking the entire 6K gene. Despite the fact that multiple attempts with the Δ6K cDNA clone (pSAV3-HH Δ6K) were made to recover virus both from transfected CHH-1 and CHSE-214 cells, CPE was not observed and no virus was recovered. We next examined whether viral proteins were expressed in transfected cells by immunostaining (Figure 6). CHH-1 cells were transfected with either full-length SAV3 (pSAV3-HHFL) or SAV3 lacking 6K (pSAV3-HHΔ6K) cDNA clones and expression of viral proteins was examined at 2, 3, 4, 6, 8, 10 dpt. Due to complete CPE present at day 8 following transfection with full-length SAV, these cells were examined for 6 days. At 2 dpt, viral protein expression was observed both in full-length (Figure 6A) and Δ6K cDNA transfected cells (Figure 6E). At 3 dpt, a distinct difference of viral protein expression was seen between full-length SAV3 (Figure 6B) and Δ6K mutant (Figure 6F). The number of positive cells was higher for CHH-1 cells transfected with full-length SAV, and the staining of virus-positive cells was shown in a multi-focused pattern indicating spread of virus from primary infected cells to neighboring cells. At 4 dpt, a large number of virus-positive cells were seen for pSAV3-HHFL transfected culture and correspondingly, changes in cell morphology started to appear (Figure 6C). By day 6 the virus infection had spread to all cells and evident CPE was present (Figure 6D). In contrast, the number of virus positive cells increased slightly in CHH-1 transfected with pSAV3-HHΔ6K cDNA up to 4 dpt, waning at 8 and 10 dpt (Figure 6G–J). Correspondingly, no CPE was observed (Figure 6E–J) and the cell cultures grew to higher density at late time points (Figure 6E versus 6J). These results show that viral proteins were expressed in pSAV3-HHΔ6K cDNA transfected cells but infectious virus was not recovered spreading from the primary transfected cells.

### Structural viral proteins expressed in pSAV3-HHΔ6K transfected cells were retained in the cytoplasm and could not be detected on the cell membrane

**Figure 7:**
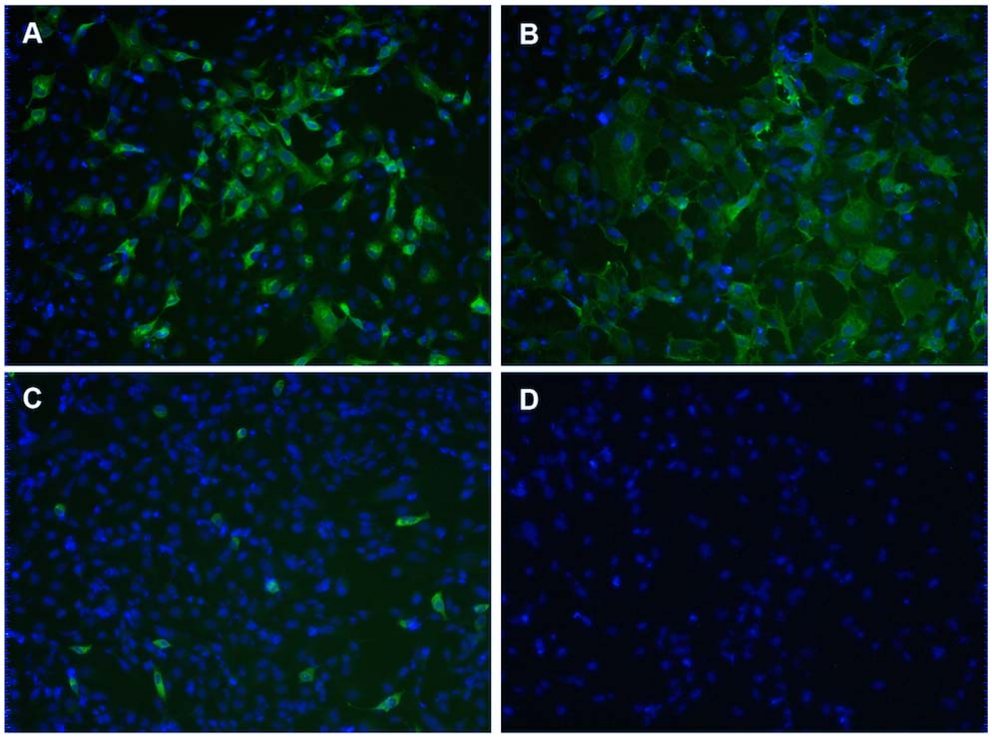
Examination of viral protein trafficking to the plasma membrane. CHH-1 cells were transfected with pSAV3-HHFL cDNA (A and B) or pSAV3-HHΔ6k cDNA (C and D). At 4 dpt, cells were immunostained under permeablized (A and C) or non-permeablized (B and D) conditions. Green fluorescence indicates localization of expressed viral proteins while blue fluorescence shows cell nucleus counterstained by DAPI. The image is representing at least three repeated experiments.

To explain the results above, we assumed that the spread of virus was impaired due to a budding defect of virus lacking 6K, which has been shown for other alphaviruses [19]. If this was the case, viral proteins should be detected on the plasma membrane of pSAV3-HHΔ6K transfected cells. To test this, transfected cells were immunostained under both permeabilized and non-permeabilized conditions. In pSAV3-HHFL transfected cells, viral proteins were clearly observed both in the cytoplasm (Figure 7A) and on the plasma membrane (Figure 7B), indicating that viral proteins were transported to the cell surface. Although viral proteins were detected in the cytoplasm of cells transfected with pSAV3-HHΔ6K (Figure 7C), we were not able to detect any viral proteins on the cell surface (Figure 7D). The finding suggests that the structural viral proteins in pSAV3-HHΔ6K transfected cells were retained in the cytoplasm and were not transported to the cell membrane.

### SAV3 lacking the entire 6K yielded an E2 protein of larger size

**Figure 8:**
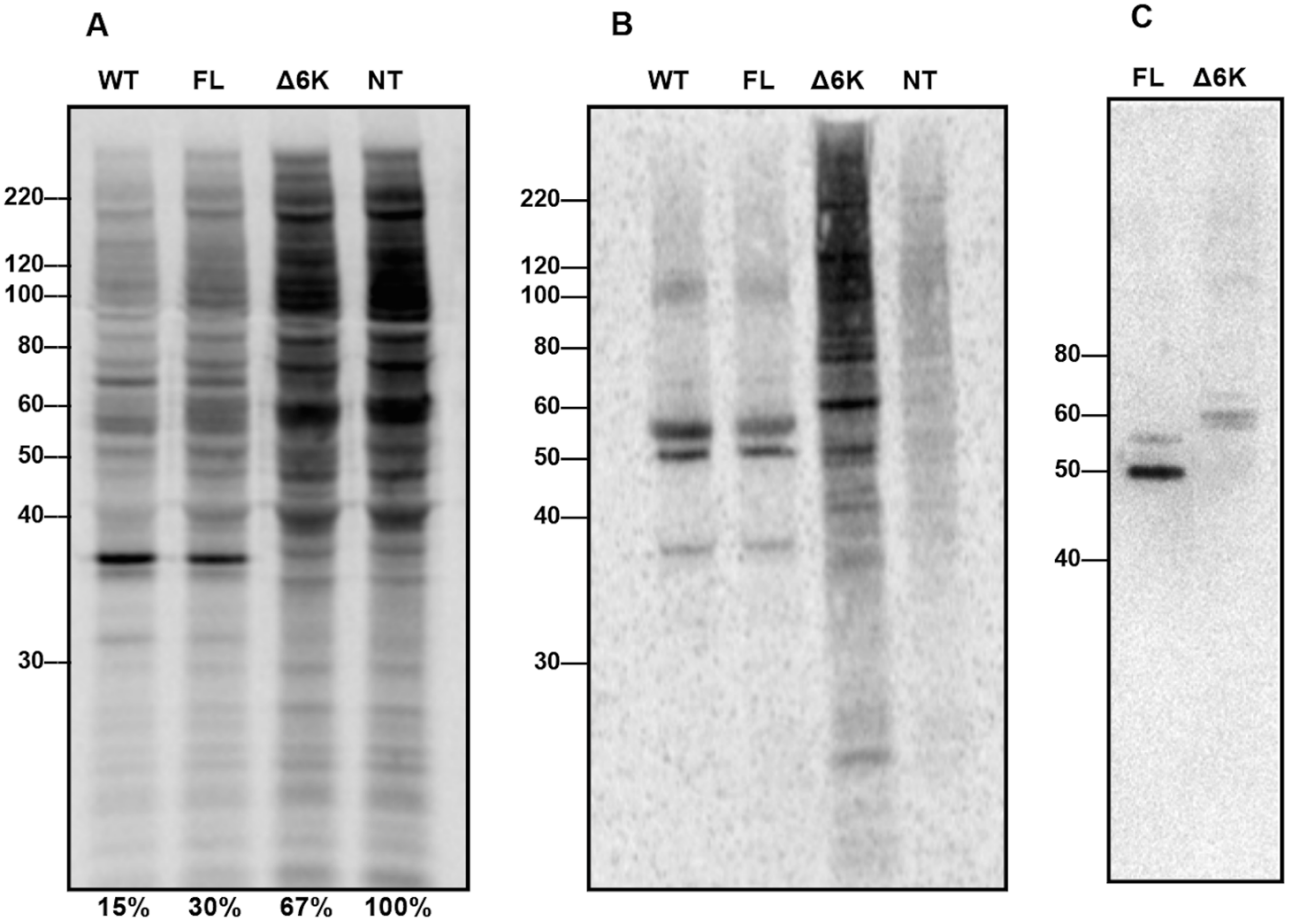
Analysis of viral proteins expression by RIPA and western blot. (A) SDS-PAGE and autoradiography of S^35^ labeled total proteins from cell lysates of CHH-1 cells infected with wtSAV3 (WT), transfected with pSAV3-HHFL cDNA (FL), or transfected with pSAV3-HHΔ6k cDNA (Δ6k). Non-treated (NT) cells were used as protein expression control. The protein density of each transfected group relative to the control group (set as 100%) is noted under the graph. (B) Total proteins from cell lysates were further immunoprecipitated using the anti-SAV3-SP antibody and revealed several bands corresponding to SAV3 viral proteins. The pattern of viral proteins from wtSAV3 infected and pSAV3-HHFL transfected cells was identical while being different in pSAV3-HHΔ6k transfected cells, where a strong band slightly larger than 60 kDa was identified. (C) Immunopreciptated viral proteins were also examined by Western blot using rabbit anti-SAV3-E2 antibody and again one band slightly larger than 60 kDa was found in line with the result from (B) indicating suboptimal processing of E2.

Since alphaviruses produce polyproteins which are subsequently cleaved into separate proteins, we wanted to know whether viral proteins were properly processed in pSAV3-HHΔ6K transfected cells. To study viral protein synthesis, we metabolically labeled the newly synthesized proteins with S^35^ methionine. Characterization of total proteins by SDS-PAGE and autoradiography showed a partial shutdown of protein translation in both wt SAV3 infected (85% shutdown) and pSAV3-HHFL cDNA transfected cells (70% shutdown). In contrast only a moderate shutdown (33%) was seen in pSAV3-HHΔ6K cDNA transfected cells (Figure 8A). Newly synthesized SAV viral proteins, one being the putative capsid protein with the size of around 37 kDa was also visualized by autoradiography, which is in agreement with the size of the capsid protein reported earlier by Moriette et. al [31]. However, in contrast to other alphaviruses, the fact that SAV does not induce total shutdown of host protein translation makes it difficult to identify each single viral protein or polyprotein. In order to enrich viral proteins over host proteins, S^35^ labeled total proteins were separated into an aqueous and a detergent phase and radioimmunoprecipitated with mouse anti-SAV3-SP antibody against structural proteins. The separated detergent phase was applied to RIPA as it is assumed that most SAV3 viral structural proteins will be located either on ER/Golgi or plasma membranes. The results showed virus-specific bands (Figure 8B), similar to the viral proteins detected by western blot using mouse anti-SAV3-SP antibody (Figure 3C). For the wt SAV3 and full-length construct a faint capsid protein band was still present in the membrane fraction, very likely representing preassembled virus particles found on membranes [32]. As mentioned above, proteins corresponding to approximately 50 kDa and 57 kDa were possibly E2, E1 and pE2 (E2+E3). It is noticeable that the viral protein patterns for wt SAV3 and rSAV3 were identical (Figure 8B). The protein pattern in pSAV3-HHΔ6K cDNA transfected cells was however different. One strong band with a molecular mass slightly larger than 60 kDa was found only in pSAV3-HHΔ6K transfected cells and not present in wt SAV3 infected and pSAV3-HHFL cDNA transfected cells (Figure 8B). Western blot analysis using the rabbit anti-SAV3-E2 specific antibody [22] confirmed that this protein was indeed virus specific (Figure 8C), likely corresponding to a precursor of the E2 protein, but cannot be the non-cleaved E2–E1 as the size was too small.

### The infectivity of SAV3 lacking the entire 6K was rescued through viral RNA recombination

**Figure 9:**
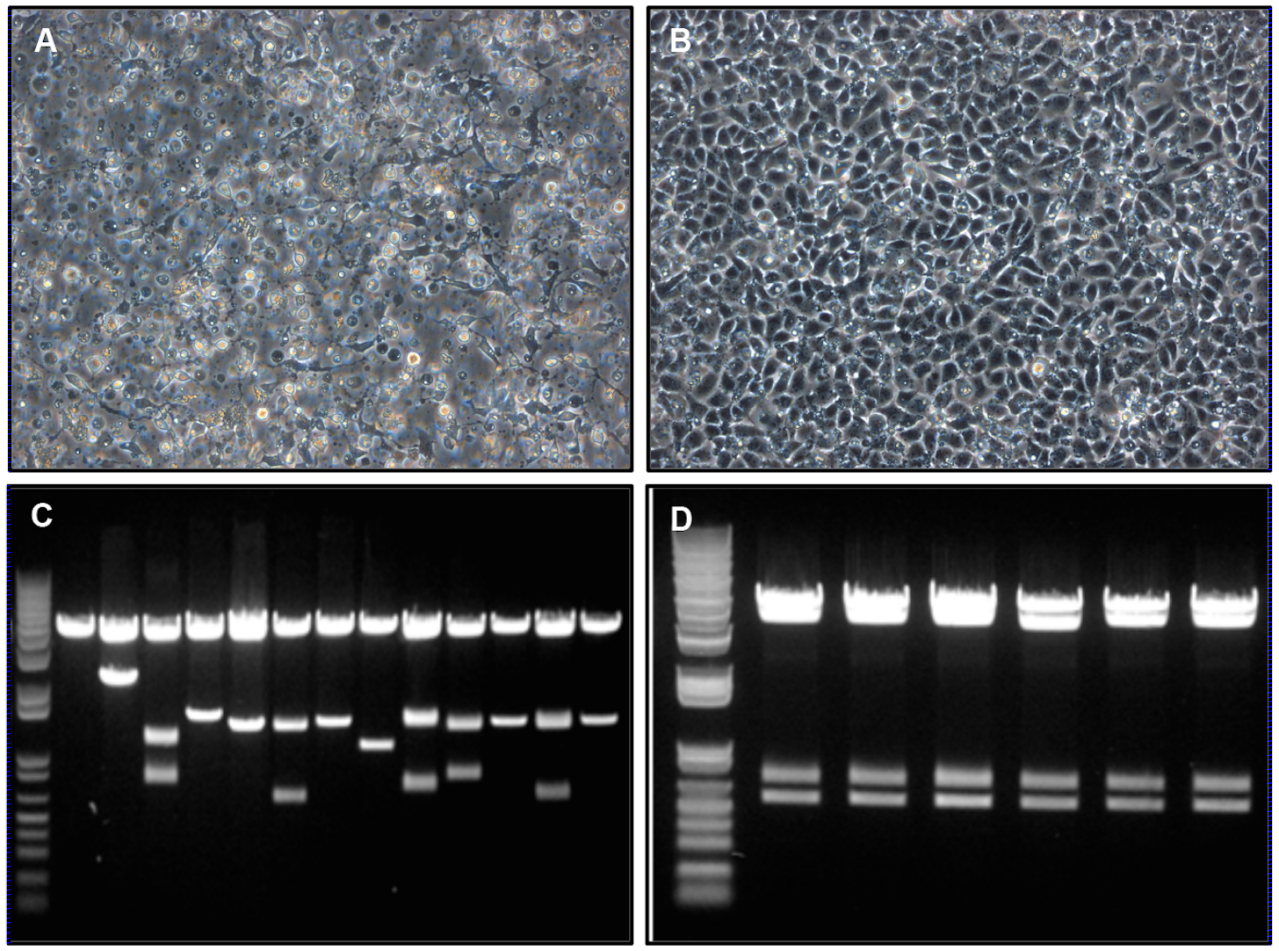
SAV3 RNA recombination and sequence variation. Extensive CPE appeared after co-transfection of pSAV3-HHΔ6k and the helper cDNA construct in CHH-1 cells. From a total of 48 parallel wells monitored, 14 wells were CPE-positive (A) and 34 wells were CPE-negative (B). (C) Supernatant from a well showing CPE were collected. SAV structural genes were amplified and cloned, before plasmids from 12 clones were enzyme digested with *EcoR*I and *BsrG*I (Lane 2–13). Lane 1 is the empty backbone plasmid. Three fragments per lane indicated by arrows show presence of the 6K gene (in 5 out of 14 clones). Fragments of different sizes suggested that recombination did not occur at precise sites. (D) Analysis of virus supernatant from the first passage. SAV structural genes were amplified and cloned, before plasmids from 6 clones were enzyme digested with *EcoR*I, *EcoR*V, and *BsrG*I. Four fragments (in all lanes) indicate the presence of 6K gene, and identical patterns among all clones suggest selection of viable recombinations in the first passage.

To investigate whether deficient production of progeny from SAV3 lacking 6K could be rescued by providing a helper plasmid, the pSAV3-HHΔ6K cDNA was co-transfected with a helper cDNA containing the whole structural gene region in CHH-1 cells. At 12 dpt, CPE was detected in 14 out of 48 transfected wells (Figure 9A&B). To examine whether viruses obtained from these CPE-positive wells were fully infectious or propagation-defective that undergo only one round of infection, virus supernatant derived from the transfected cells was passaged to new CHH-1 cells. Development of CPE was consistently seen for three passages, demonstrating successful formation of infectious virus likely through RNA recombination during viral replication. To prove this, supernatant was collected from a well showing CPE and from which viral RNA was isolated and the SAV structural genes were amplified from the capsid protein gene to E1. After cloning and transformation, plasmids were extracted from 12 colonies and digested with the restriction endonucleases *EcoR* I and *BsrG* I. As *BsrG* I is a unique site within 6K, digested plasmids will contain three fragments if 6K is present, otherwise only two fragments will appear (Figure 9C). The results showed that 5 out of 12 examined clones contained the 6K gene, which was further confirmed by DNA sequencing. Notably, these clones contained fragments of different sizes, suggesting that recombination did not occur at precise sites. We therefore went on to study what was present in the following passage. Viral RNAs isolated from the first passage were amplified by PCR targeting the region between nsP4 and E1. Following cloning and digestion with *EcoR* I, *EcoR* V, and *BsrG* I, plasmids will contain four fragments if the 6K gene was present. The result showed that all digested clones contained 6K in the viral genome (Figure 9D). One of the cloned viral genomes was fully sequenced and the result revealed the genome sequence identical to that of parental virus (SAV3-H10).

## Discussion

This is the first report to document the importance of 6K for the generation of infectious progeny of a salmonid alphavirus, and SAV3 lacking the entire 6K protein was not able to form infectious particles. Although viral proteins were expressed within pSAV3-HHΔ6K transfected cells, viral envelope proteins were not detected on the cell surface and no viable virus was released from transfected cells that could spread to neighboring cells. In contrast, cell-to-cell spread from initial transfected cells was observed following transfection with the full-length SAV3 construct (pSAV3-HHFL).

Studies of other alphaviruses have shown that 6K is dispensable for infection as a variant lacking the entire 6K still formed infectious viral particles, although infectivity was attenuated and virus budding was affected leading to decreased virus titers [18], [19]. In contrast, our results show that SAV is sensitive to 6K-deletion. Furthermore, we found that SAV contains a unique seven-amino acid domain “GVRGWSA”, conserved within the three fully sequenced subtypes SAV1–3, and not found in other alphavirus species. The 6K protein is associated with membranes and it is not unlikely that this insertion adapts the 6K protein to function in cold-blooded animal species that carry a different membrane composition compared to warm-blooded animals. To maintain the viscosity of their membranes as poikilothermic animals, fish cell membranes contain a high content of unsaturated fatty acids to keep them fluid at low temperatures. What role this unique domain in SAV3 6K plays for infectivity will be investigated in the future.

Although there are functional differences, similarities exist between the 6K gene of SAV3 and other alphaviruses. This includes conservation of an aromatic domain, despite one key residue, a non-aromatic amino acid glycine in position 19 of SAV1–, differed from the aromatic amino acid tryptophan found in other alphaviruses (Figure 5A). Furthermore, hydrophobicity pattern and two putative transmembrane domains of SAV separated by a cysteine-rich region also share similarities with other alphaviruses. All these similarities theoretically imply that SAV3 6K functions as a viroporin in a similar way as for other alphaviruses, while being indispensable for SAV3 infectivity. This is comparable to the viroporin p7 protein of HCV which is also essential for infectivity [33], [34] and viability [35]. Due to this, p7 has become an interesting target for development of antiviral compounds [36]–[39]. Likewise, our findings suggest that targeting 6K of SAV3 might be an antiviral approach.

Despite 6K provides cleavage sites for polyprotein processing, deletion of the entire 6K gene of SFV had no impact on polyprotein processing, E1/E2 heterodimerization, and intracellular trafficking [18]. Proper glycoprotein proteolytic processing was however hampered in SINV variants containing either a partially deleted 6K lacking the amino acid residuals 24 to 45 or an in-frame insertion of 15 amino acid within the 6K gene, resulting in reduced formation of viral particles and reduced plaque size [20], [40]. We could not unequivocally determine whether the process of SAV polyprotein cleavage shares similarity to other alphaviruses, partly because protein shutdown was not 100% in the host cells, making it difficult to identify the viral proteins during protein synthesis. To resolve this problem, development of antibodies specifically recognizing each viral protein will be needed. When we compared the size of the E2 glycoprotein of the Δ6K mutant by radioimmunoprecipitation and western blot, we observed that the glycoprotein was of larger size than E2 of wild-type SAV3. Impaired polyprotein processing between E2 and E1 leading to the production of unauthentic E2 proteins could explain this. Another explanation is that the large size of E2 of the Δ6K mutant could result from incorrect post-translational modification, appearing as a heavily non-trimmed glycosylated protein stuck in the secretory pathway. Although immunofluorescent staining showed that viral proteins were expressed within pSAV3-HHΔ6K transfected cells, viral proteins were not detected on the cell surface, suggesting impaired trafficking of viral proteins from the ER or other intracellular compartments to the plasma membrane.

Non-segmented RNA viruses, such as picornaviruses, coronaviruses, alphaviruses and several plant viruses, accelerate genome exchange through RNA recombination [41]. In this study, we demonstrated frequent RNA recombination by SAV3 within cells transfected with pSAV3-HHΔ6K cDNA together with a helper cDNA clone containing all structural genes including 6K gene in CHH-1 cells. A recent publication by our group documenting that natural infection of SAV3 generates numerous viral deletion mutants through imprecise RNA recombination [42], is in agreement with the result of this study showing recombination in co-transfected cells in vitro at non-specific sites. After one round of passage in cells, we found that the structure of all plasmids analyzed by restriction enzyme digestion was identical, documenting strong selection for viable variants. The fact that RNA recombination occurs in alphaviruses therefore seems to address pivotal safety concerns regarding recombinant alphaviruses used as attenuated vaccines.

SAV subtypes 1–3 are closely related but differences within the nsP3 gene consists of several insertion/deletion sequences, likely have arisen through RNA recombination [4], [43]. The mechanisms of recombination of non-segmented genomes are not fully understood, but occur during viral replication and likely involve switching between viral templates while holding on to the nascent viral strand [44]. More detailed studies of alphavirus RNA recombination has been performed in SINV [45]–[47], showing that RNA recombination of SINV gives rise to deletions, insertions and genome rearrangements [48]. Interestingly, many of these altered SINV RNAs are still infectious. Similarly, Hahn et al. [49] demonstrated that WEEV is a recombinant virus and suggested that WEEV appears to have arisen by recombination between a EEEV-like virus (capsid protein) and a SINV-like virus (glycoproteins), which underlines the importance of RNA recombination in virus evolution.

For more than one decade, CHSE-214 and BF-2 cells have been widely used for propagation of SAV [2], [4], [43], [50]. The CHH-1 cell line develops CPE faster than CHSE-214 and Salmon head kidney-1 (SHK-1) cells after infection with SAV1 [51]. Based on the above, we compared recovery of recombinant SAV3 in these three cell lines, CHSE-214, BF-2 and CHH-1. In conformity with the published results, CPE occurred earlier after transfection with the full-length cDNA clone in CHH-1 cells compared to CHSE-214 and BF-2 cells. Moreover, a 10 times higher virus titer was obtained in CHH-1 cells. As SAV is highly sensitive to IFN-α responses [22], the difference was likely due to CHH-1 cells generating weak IFN-α and ISGs responses to SAV3 infection when compared to the other two cells lines (Figure 4). Similarly, Baby hamster kidney-21 (BHK-21), an IFN-deficient cell line, has been widely used for the production of high titer alphaviruses [52], [53].

We have also shown that a SFV replicon construct containing the SAV3 structural genes (capsid-E3–E2-6K–E1) delivered as a DNA vaccine to mice for primary immunization and combined with E2 recombinant protein boosting elicited production of high titer anti-SAV3 antiserum, as shown by immunostaining of cells, immunohistochemistry, and western blot. However, chimeric alphaviruses composed by a SFV replicon and SAV3 structural proteins failed to assemble into virus particles in transfected BHK-21 cells (no CPE) despite high levels of SAV3 structural proteins being detected in the cytoplasm (data not shown). It is possible that the SAV3 capsid protein does not recognize the SFV packaging signal located in nsP2 [54] or that surface proteins of SAV3 (E1 and E2) adapted for cold-water environment are not functional at higher temperatures.

Based on the finding of SFV lacking the entire 6K gene giving rise to the formation of the attenuated virus, we were interested in exploring if deletion of the SAV3 6K gene could similarly attenuate virus infectivity and constitute a strategy for a live-attenuated viral vaccine. This was not a viable strategy. Future studies addressing 6K partial deletion variants in detail or including co-transfection with a plasmid encoding 6K in trans for virus rescue should be conducted. Finally identification of an IFN-α deficient cell-line supporting growth of salmonid alphaviruses will be of great importance for rescue of attenuated SAV variants and for production of high virus titers.
